# Climate-Sensitive Redistribution of Veterinary Parasites: An Attribution Framework for One Health Surveillance and Control

**DOI:** 10.3390/biology15181576

**Published:** 2026-09-08

**Authors:** Abel Villa-Mancera, José Manuel Robles-Robles, Jaime Olivares-Pérez, Agustín Olmedo-Juárez, Alejandro Córdova-Izquierdo, Roberto González-Garduño, José Luis Ponce-Covarrubias, Nallely Rivero-Perez, Felipe Patricio, Huitziméngari Campos-García, Maria José Robles-Rosado, Juan Ricardo Cruz-Aviña, Samuel Ortega-Vargas

**Affiliations:** 1 Facultad de Medicina Veterinaria y Zootecnia, Benemérita Universidad Autónoma de Puebla, Tecamachalco 75482, Puebla, Mexico; manuel.roblesr@correo.buap.mx (J.M.R.-R.); felipe.patriciomtz@correo.buap.mx (F.P.); huitzi.campos@correo.buap.mx (H.C.-G.); juan.cruzavina@correo.buap.mx (J.R.C.-A.); samuel.ortega@correo.buap.mx (S.O.-V.); 2 Unidad Académica de Medicina Veterinaria y Zootecnia, Universidad Autónoma de Guerrero, Ciudad Altamirano 40662, Guerrero, Mexico; jaimeolivares@uagro.mx; 3 Centro Nacional de Investigación Disciplinaria en Salud Animal e Inocuidad (CENID SAI-INIFAP), Jiutepec 62550, Morelos, Mexico; olmedo.agustin@inifap.gob.mx; 4 Departamento de Producción Agrícola y Animal, Universidad Autónoma Metropolitana, Unidad Xochimilco, CDMX 04960, Mexico; acordova@correo.xoc.uam.mx; 5 Unidad Regional Universitaria Sursureste, Universidad Autónoma Chapingo, Teapa 86800, Tabasco, Mexico; rgonzalezg@chapingo.mx; 6 Escuela Superior de Medicina Veterinaria y Zootecnia No. 3, Universidad Autónoma de Guerrero, Técpan de Galeana 04960, Guerrero, Mexico; jlponce@uagro.mx; 7 Área Académica de Medicina Veterinaria y Zootecnia, Instituto de Ciencias Agropecuarias, Universidad Autónoma del Estado de Hidalgo, Pachuca de Soto 40900, Hidalgo, Mexico; nallely_rivero@uaeh.edu.mx; 8 Posgrado en Socio Economía, Estadística e Informática-Desarrollo Rural, Colegio de Posgraduados, Campus Montecillo, Texcoco 56264, Estado de México, Mexico; robles.rosado@colpos.mx

**Keywords:** climate change, veterinary parasitology, parasite redistribution, ecological attribution, tiered surveillance, One Health, microclimate, refugia, anthelmintic resistance

## Abstract

Climate change is reshaping veterinary parasite transmission by altering thermal and hydrological suitability, environmental stage persistence, vector and intermediate host ecology, and contact across livestock–wildlife–companion animal interfaces. These effects are nonlinear; while warming may extend transmission in some systems, heat, desiccation, habitat loss, or disrupted hydrology can reduce the risk or concentrate transmission in local refugia. This critical narrative review compares pasture-transmitted helminths, snail-borne trematodes, environmentally transmitted protozoa, vector-borne parasites, and multi-host cycles. We propose an attribution framework that classifies observed changes across four dimensions (geographic range, seasonal timing, transmission intensity, and host-interface structure) and evaluates them through five analytical filters: suitability, parasite life-cycle response, vector or intermediate-host response, host-interface change, and surveillance artifacts. This framework prevents improved detection, land-use change, animal movement, management shifts and improved detection from being mistaken for climate-driven emergence. We also propose a climate–refugia paradox hypothesis, requiring empirical validation, in which drought or heat may reduce unselected parasite refugia and intensify selection for anthelmintic resistance. Finally, we connect a tiered diagnostic approach from field tools to reference molecular surveillance to support attribution-aware, risk-based One Health strategies that protect animal production, biodiversity, and public health.

## Simple Summary

Climate change does not affect all veterinary parasites equally. In some regions, warming may extend the transmission seasons or allow parasites and their vectors to move into new areas. In contrast, extreme heat, drought, habitat loss, or altered water availability may reduce transmission or concentrate it in small local habitats. This review explains how climate can redistribute parasite risk across places, seasons, animal populations, and contact zones, linking livestock, wildlife, companion animals, and humans. It proposes a practical framework for distinguishing genuine climate-related changes from effects caused by animal movement, land-use change, improved diagnostics, or more intensive surveillance. The review also proposes a climate–refugia hypothesis requiring empirical validation: when drought or heat reduces environmental parasite stages and refugia, whole-group treatment could increase the proportion of surviving parasites exposed to drug selection. Finally, a tiered surveillance approach that links affordable field methods with regional confirmation and advanced molecular tools is presented. This framework can support more accurate surveillance, better-targeted control, and stronger preparedness for One Health.

## 1. Introduction

Parasitic diseases remain a persistent constraint on animal health, livestock productivity, wildlife conservation and rural livelihoods [1]. The epidemiology of these diseases is closely associated with the environment, as many parasites require specific combinations of temperature, humidity, rainfall, vegetation, soil moisture, aquatic habitats, vectors, and intermediate hosts to complete their life cycles [2]. Climate change is reorganizing these conditions across space and time. Some parasites may gain new ecological opportunities, whereas others may lose their suitable habitats [3]. Therefore, the central question is not simply whether climate change will increase parasitism but how it will redistribute the risk among regions, seasons, host species, production systems, and wildlife–livestock interfaces.

Vector-borne parasite transmission is particularly sensitive to climate change because temperature, precipitation, humidity, and seasonality influence vector survival, reproductive rates, host-seeking activity, seasonal abundance, and geographic distribution [4,5]. Consequently, the risk landscapes of babesiosis, theileriosis, trypanosomiasis, leishmaniasis, and filarial infections may shift. However, climate is only part of the explanation; land use, irrigation, livestock movement, shifts in wildlife distribution and wildlife–livestock contact, acaricide selection pressure, and socioeconomic vulnerability often determine whether climatic suitability results in sustained transmission [6,7]. Therefore, regional, mechanistic, and surveillance-based analyses are more informative than broad global projections.

Wildlife may function as reservoir or bridge hosts, spillover recipients, or ecological sentinels, whereas parasites can regulate host populations, alter species interactions, and shape biodiversity [8,9,10]. Climate-related changes in wildlife distribution, migration, and resource use may alter their contact with livestock, especially in rangelands, wetlands, forest edges, protected areas, and peri-urban zones, where drought or habitat loss concentrates animals around shared resources [11].

Despite the rapid growth of literature on climate change and infectious diseases, several weaknesses remain in veterinary parasitology. Many studies rely on cross-sectional prevalence data and broad climatic averages, making it difficult to distinguish persistent redistribution from short-term epidemiological fluctuations [12]. Microclimatic conditions that directly regulate parasite development, such as soil moisture, vegetation cover, shade, water permanence, dung humidity, and snail habitat, are often poorly measured [13]. Diagnostic heterogeneity can further distort apparent climate associations when coprology is compared with serology, polymerase chain reaction (PCR), quantitative PCR (qPCR), sequencing, and antigen detection [14]. Wildlife and intermediate hosts remain underrepresented in predictive models, and tropical and subtropical regions, where parasite diversity and climate vulnerability are often greatest, continue to be under-surveilled [12].

We argue that climate change reorganizes the ecological architecture of veterinary parasitism rather than simply increasing its prevalence. The main contribution of this review is a proposed attribution framework in which suspected redistribution is first classified as geographic, seasonal, intensity-related, or interface-related, and then examined using five mechanistic and surveillance filters. The framework is intended to guide what should be sampled, which biological state should be measured, when diagnostics should be escalated, and how confidently a signal can be attributed to climate rather than land use, animal movement, management, or changing detection effort.

The review is organized around three questions. Which biological and ecological mechanisms make veterinary parasites responsive to climate variability and long-term changes? How do these mechanisms differ among parasites transmitted through pasture stages, aquatic intermediate hosts, resistant environmental stages, arthropod vectors, and multi-host cycles? Finally, which diagnostic, surveillance, and control systems can detect and manage redistribution without conflating climatic effects with competing drivers? Throughout, the relevant unit of analysis was the complete transmission system rather than host prevalence alone.

## 2. Review Approach and Conceptual Scope

This article was developed as a critical narrative review, rather than a systematic review or meta-analysis. Relevant literature was identified through iterative searches of PubMed/MEDLINE and targeted publisher-platform searches in ScienceDirect (Elsevier), SpringerLink, Wiley Online Library, Taylor & Francis Online, Cambridge Core, Frontiers, MDPI, and Nature Portfolio. Searches were supplemented by backward citation tracking and targeted retrieval of recent reviews, mechanistic studies, longitudinal surveillance, predictive models, and diagnostic advances. No lower publication-date limit was imposed; searches were updated through July 2026. No formal language restriction was applied, although the final synthesis was dominated by English-language peer-reviewed literature. Literature selection was purposive rather than exhaustive and prioritized studies with direct relevance to veterinary parasites and climate-sensitive transmission, particularly mechanistic, longitudinal, experimental, molecularly resolved, and independently validated modeling evidence. Cross-sectional studies were retained when they illustrated important regional, host, or diagnostic gaps but were not treated as evidence of climate causation on their own.

The synthesis prioritized evidence that clarified mechanisms or attributions, including longitudinal datasets, experiments on temperature, moisture, or host responses, field studies integrating microclimate, hydrology, vector ecology, or intermediate host ecology, molecularly resolved transmission studies, and models that represented parasite biology or quantified uncertainty. Cross-sectional prevalence reports were retained when they illustrated important host, regional, or diagnostic gaps, but were not treated as evidence of climate causation on their own.

Throughout this review, we use the term ‘climate-sensitive redistribution.’ It acknowledges that climatic conditions can alter ecological suitability without implying that every change in range or prevalence is caused by anthropogenic climate change. Therefore, climatic effects are considered alongside land use, irrigation, animal movement, host density, management, vector control, drug resistance, and changes in diagnostic effort.

Because the evidence spans diverse parasite groups and study designs, we did not calculate pooled estimates or conduct a formal risk-of-bias assessment. Instead, the review compares transmission architectures, identifies where causal inference is strongest or weakest, and translates the evidence into an operational attribution matrix, a tiered diagnostic framework, and principles for climate-adaptive One Health control strategies.

## 3. Mechanisms of Climate-Sensitive Parasite Redistribution

### 3.1. Thermal and Hydrological Constraints

Climate-sensitive redistribution emerges from the interactions among thermal, hydrological, ecological, and host processes [15,16]. Warming can accelerate development, shorten generation times, or prolong vector activity; however, excessive heat and desiccation may suppress these environmental stages [17,18]. Rainfall can promote larval migration and create snail habitats, whereas intense events may disperse infective stages, alter grazing patterns, or increase runoff. Drought may reduce transmission across open pastures while concentrating hosts, vectors, and intermediate hosts around scarce water sources [15,16].

The balance among these processes differs between transmission systems. Ectothermic parasite stages and arthropod vectors respond directly to temperature; however, transmission also depends on moisture, breeding habitat, competent hosts, and management. Therefore, warming may support poleward or upslope activity of ticks, mosquitoes, or flies only where humidity and habitat remain suitable; once physiological thresholds are exceeded, survival falls [19,20,21].

Moisture strongly influences the persistence and movement of helminth larvae [22,23] and environmentally resistant protozoan stages [24]. In arid and semi-arid landscapes, the regional climate may appear unsuitable, even though irrigation canals, leaking infrastructure, shaded pastures, poorly drained soil, and artificial water points sustain high-risk microrefugia [25,26]. Thus, redistribution may take the form of focal persistence or concentration within an otherwise hostile landscape rather than a broad geographic expansion [23,25,26]. Examples include irrigation networks that maintain lymnaeid snail habitat for *Fasciola* transmission and shaded or irrigated pasture patches that retain moisture and support infective gastrointestinal nematode larvae despite a dry surrounding landscape.

### 3.2. Extreme Events and Temporal Redistribution

Droughts, floods, heatwaves, storms, and unusual seasonal transitions can abruptly alter host aggregation, environmental contamination, vector abundance, intermediate host habitats, and management [27]. Annual averages often obscure these short-lived anomalies, even when determining whether a parasite completes its life cycle [28]. Heat stress can also affect host immunity, body condition, grazing, housing, water use, and movement, creating exposure patterns that temperature averages alone cannot capture [29,30].

Earlier springs, warmer autumns, irregular rainy seasons, and shorter dry periods may shift the onset, peak, and duration of transmission without changing the annual prevalence [16,18]. These phenological shifts may reduce the effectiveness of fixed control calendars by altering the periods of the greatest environmental contamination, host exposure, and infection risk [17,18]. Climate-driven changes in the timing of wildlife migration and reproduction may also modify temporal overlap with livestock, thereby increasing or decreasing opportunities for cross-species transmission [31,32]. In veterinary practice, such shifts can move the optimal timing of targeted anthelmintic treatment away from historical gastrointestinal-nematode peaks and can require acaricide decisions to follow current tick activity rather than a fixed seasonal calendar.

### 3.3. Microclimate, Land Use and Host Movement

Annual temperature, total precipitation, and broad climate classes are useful for regional comparisons, but they may not reflect the microhabitats experienced by parasites. Transmission can persist in dung, shaded vegetation, leaf litter, riparian strips, small water bodies, or host resting sites, even when the surrounding macroclimate appears unsuitable, and vice versa. Therefore, predictive models should combine remote sensing with ground-level measurements of microclimate, hydrology, vegetation, host density, and parasite occurrence [15,20,21,25].

Climate rarely acts independently of deforestation, irrigation, agricultural expansion, urbanization, habitat fragmentation, or livestock intensification. These processes create new ecotones and alter moisture, vector communities, intermediate hosts, and contact with vertebrate hosts. Therefore, attribution must consider climate together with land use, management, diagnostic method, and control history, rather than treating a climatic correlation as evidence of causation [20,27,28,31].

Host movement is a pathway of redistribution. Drought, pasture scarcity, flooding, heat stress, trade, and seasonal grazing can displace livestock, whereas wildlife may shift their range or gather around the remaining resources. Such movements can introduce parasites into new areas, expose naïve hosts to parasites, and reorganize wildlife–livestock–companion animal interfaces. Therefore, surveillance needs to integrate hosts, vectors, and environmental compartments rather than examining them separately [11,31]. Moving naïve livestock into an endemic area can precipitate severe clinical disease when exposure is intense, whereas moving infected animals into a climatically receptive area may introduce parasites and initiate local transmission.

### 3.4. From Association to Ecological Attribution: A Proposed Operational Decision Framework

A central challenge is the transition from statistical association to ecological attribution [27,33]. A correlation between parasite occurrence and temperature or rainfall is insufficient to demonstrate a climatic effect, particularly when surveillance intensity, diagnostic sensitivity, land use, host movement, irrigation, treatment history, or vector control practices also vary across space or time [15,33]. Stronger attribution requires repeated or longitudinal observations combined with mechanistic, experimental, or quasi-experimental evidence linking the epidemiological signal to a biologically plausible process, while explicitly evaluating confounding variables and competing explanations [27,28,33].

For operational purposes, we propose a two-step framework for evaluating climate-sensitive parasite redistribution. The first step classifies the observed signal along four non-mutually exclusive dimensions: (1) geographic redistribution, including range expansion, contraction, or displacement; (2) seasonal redistribution, reflected in changes in the onset, peak, duration, or overwintering of transmission; (3) redistribution of transmission intensity, encompassing changes in the force of infection, parasite burden, shedding, vector infection, or clinical impact; and (4) interface redistribution, involving changes in transmission among livestock, wildlife, companion animals, vectors, intermediate hosts, and environmental compartments [27,28,34]. A single study may identify signals in more than one dimension; however, each dimension should be evaluated using a prespecified epidemiological or ecological endpoint and an explicit counterfactual comparator representing the expected pattern in the absence of the proposed climatic effect [33]. For example, a climatic attribution would be strengthened if transmission increased in a region experiencing warming while a comparable region with similar land-use and livestock-movement changes but stable microclimatic conditions did not show the same increase.

**Figure 1:**
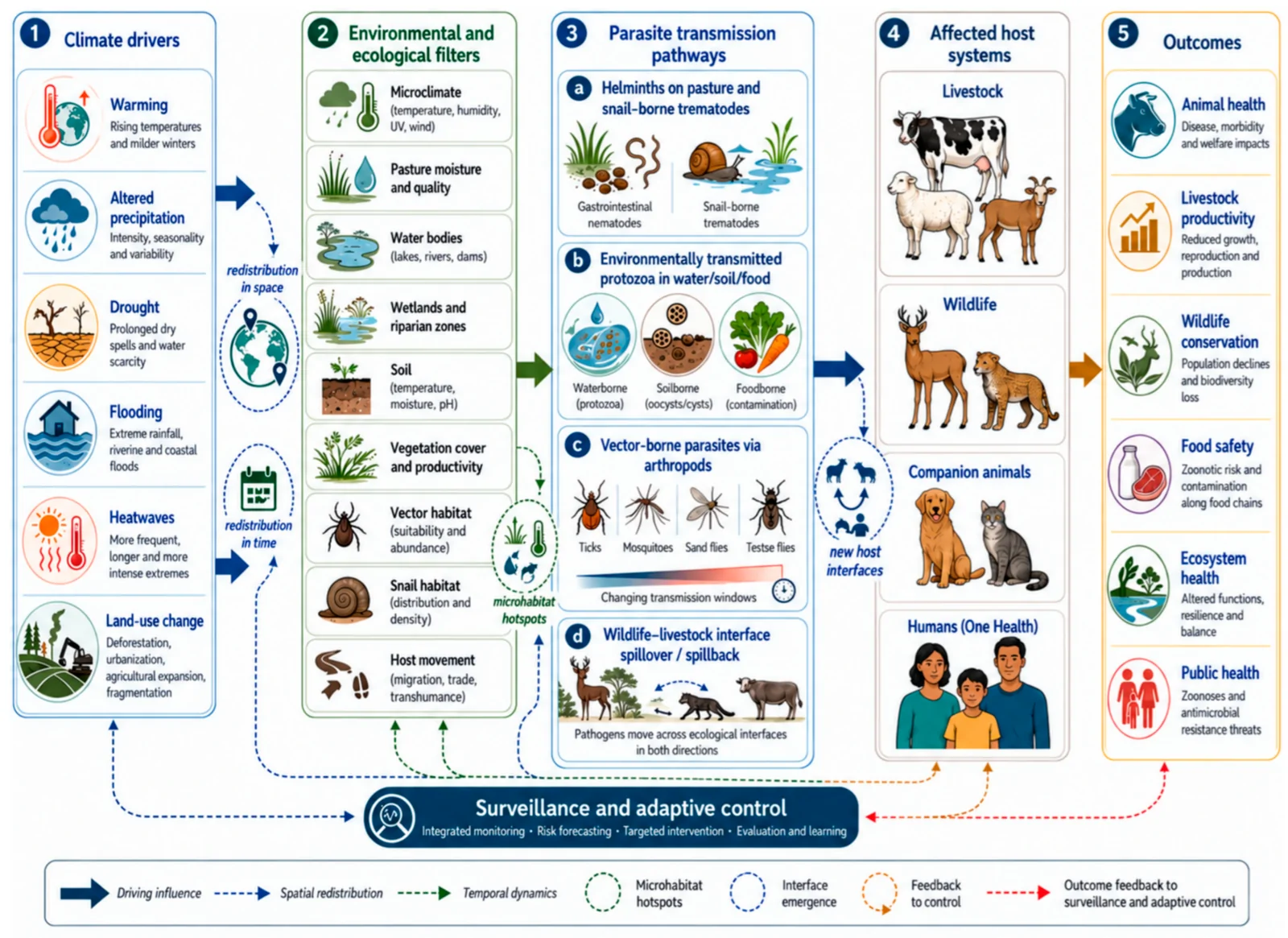
Conceptual framework for climate-sensitive redistribution of veterinary parasites. Climatic forcing acts through ecological filters, parasite life-cycle responses, vectors or intermediate hosts, host interfaces, and surveillance processes to alter the four observable dimensions of redistribution.

The second step applies a five-domain attribution filter. Depending on the parasite life cycle and transmission system, investigators should assess whether the observed signal is supported by evidence of: (1) altered environmental suitability within the microhabitat relevant to transmission; (2) a measurable change in parasite development, survival, fecundity, infectivity, or phenology; (3) a corresponding response in vectors or intermediate hosts, where applicable; (4) altered host movement, contact structure, or wildlife–livestock–companion animal interfaces; and (5) exclusion or adequate control of alternative explanations, such as intensified surveillance, diagnostic substitution, sampling bias, changes in treatment or vector-control practices, and differential reporting [27,33,34]. Together, the four dimensions and five attributional domains translate the pathways illustrated in Figure 1 into a practical workflow for evaluating climate-sensitive parasite redistribution and prioritizing diagnostic and surveillance responses.

The four redistribution dimensions and their integration with the five attribution domains are newly proposed in this review. The individual concepts are not presented as new causal principles; rather, they adapt and integrate established ideas from climate-disease ecology, counterfactual causal inference, multi-host disease ecology, and surveillance design [11,15,20,27,28,31,33]. Attribution confidence is considered low when a signal is based mainly on cross-sectional detection or climatic correlation; moderate when repeated observations show temporal and mechanistic coherence with partial control of competing explanations; and high only when longitudinal, experimental, or quasi-experimental evidence converges across relevant parasite, environmental, host, vector or intermediate-host, and diagnostic data, with alternative explanations explicitly assessed. Advanced molecular or genomic methods can strengthen source, connectivity, or species-resolution evidence but do not confer high attribution confidence by themselves. Table 1 operationalizes the four dimensions and five attributional domains, specifies the minimum evidence suggested for triggering enhanced surveillance or precautionary management, and identifies common sources of false attribution. The resulting matrix is a proposed structured decision aid rather than a validated causal instrument and requires prospective testing across contrasting climates, parasite life cycles, host communities, and transmission systems.

## 4. Redistribution Across Major Parasite Transmission Architectures

### 4.1. Pasture-Transmitted Nematodes

Pasture-transmitted gastrointestinal nematodes are climate-sensitive because their eggs and larvae develop in feces and on pastures. Temperature regulates developmental rates, while rainfall, fecal moisture, evaporation, vegetation, and local microclimate influence larval survival and migration onto herbage [18,22,35]. Because these processes operate at fine spatial and temporal scales, climatic effects are more appropriately interpreted through changes in larval development, survival, migration, and pasture infectivity than through host prevalence alone, which represents a delayed and management-dependent epidemiological endpoint [36].

In tropical and subtropical production systems, the timing and distribution of rainfall relative to preceding dry periods may be more informative than the annual mean temperature. *Haemonchus contortus* can develop rapidly when warm conditions coincide with sufficient fecal and soil moisture, whereas prolonged desiccation, high evaporation, and direct solar exposure reduce the development and survival of its free-living stages [35,37,38]. Rainfall following a dry period can restore fecal moisture, permit the development of surviving eggs and larvae, and promote the migration of infective L3 onto the herbage. These processes may generate short-lived, spatially localized post-rainfall exposure pulses, particularly in shaded or persistently moist pasture microhabitats, rather than the broader extension of the transmission season projected in some temperate regions [18,35,39]. Hypobiosis provides an additional seasonal mechanism: in *Haemonchus contortus* and other gastrointestinal nematodes, adverse seasonal cues can favor arrested larval development within the host, with resumed development contributing to later transmission or disease peaks; shifts in the timing or duration of unfavorable periods may therefore alter these patterns [17,18].

Surveillance should be aligned with rainfall pulses and include pasture and dung moisture, shade, irrigation, host aggregation, and clinical indicators, such as FAMACHA scores and body condition. In dry environments, transmission may persist in localized moist microhabitats and rebound rapidly after rainfall rather than increase uniformly throughout the year [40,41].

Climate change may also modify gastrointestinal nematode communities because genera such as *Haemonchus*, *Teladorsagia*, *Ostertagia*, *Trichostrongylus*, and *Cooperia* differ in their climatic tolerance, pathogenicity, fecundity, and anthelmintic susceptibility. Because total strongyle egg counts may conceal species replacement and changes in clinical risk, species-level surveillance using larval culture or molecular methods is recommended where feasible [22,42,43]. Where *H. contortus* increases in relative abundance or expands into new areas, its frequent association with anthelmintic resistance can compound control challenges. However, redistribution should not be assumed to imply movement of resistant genotypes without local efficacy testing or molecular evidence.

Longitudinal monitoring of wild and domestic ruminants is needed to detect persistent changes in parasite seasonality, abundance, and community composition. However, comparable long-term datasets remain limited for wildlife and tropical, subtropical, and extensive pastoral systems. Repeated standardized sampling across climatic gradients would improve comparisons among host populations and help distinguish sustained redistribution from short-term fluctuations [18].

Lungworms provide a related example of climate-sensitive transmission. The development, survival, and availability of *Dictyocaulus* larvae on pasture depend strongly on temperature and moisture, while outbreak occurrence is additionally influenced by larval dispersal, grazing exposure, and acquired host immunity [17,44]. Weather-driven changes may therefore shift the timing of peak pasture infectivity and weaken the reliability of historical seasonal expectations of infectivity. Climate-based forecasts may support targeted clinical vigilance and parasite control, although broader applications require validation beyond the regions and production systems in which the models were developed [44]. The coprophilous fungus *Pilobolus* can further enhance dispersal of *Dictyocaulus viviparus* infective larvae from dung through explosive discharge of sporangia, providing a direct biological link between pasture microclimate and larval spread [44].

Mechanistic frameworks such as GLOWORM-FL and GLOWORM-FL-DV demonstrate how weather-dependent parasite development and survival can be translated into estimates of seasonal pasture infectivity [44,45]. However, their application to new settings requires local parameterization and external validation. For operational forecasting, model outputs should also be interpreted alongside local microclimate, grazing history, host immunity, stocking patterns, and anthelmintic use.

### 4.2. Snail-Borne Trematodes

Snail-borne trematodes are highly climate-sensitive because their transmission depends on freshwater or amphibious snail intermediate hosts. *Fasciola hepatica* is generally associated with temperate and high-altitude environments, whereas *Fasciola gigantica* and many amphistomes are more common in tropical and subtropical systems [46,47]. Temperature influences parasite development within eggs and snails, whereas rainfall, flooding, irrigation, soil moisture, and persistent surface water affect snail habitat availability and seasonal transmission risk [25,26].

In tropical drylands and seasonally flooded pastoral systems, drought may reduce the overall extent of suitable snail habitats while concentrating transmission around irrigation canals, reservoirs, drainage systems, riparian margins, and permanent water points [48,49]. Subsequent rainfall or flooding may temporarily expand aquatic habitats, alter snail distribution, and increase contact between grazing animals and contaminated vegetation or water sources. Therefore, transmission may persist in localized aquatic refugia and increase rapidly after hydrological events rather than rise uniformly throughout the year [25,26]. Because the prevalence in definitive hosts cannot, by itself, reveal the environmental processes sustaining transmission, surveillance should combine animal-level diagnostics with malacological and hydrological information, including snail abundance and infection status, habitat persistence, soil moisture, irrigation and flooding patterns, and livestock access to aquatic habitats [50,51]. This integrated approach is particularly important in areas where multiple snail-borne trematodes co-circulate, including zones of *Fasciola hepatica*–*Fasciola gigantica* overlap and settings where fasciolids and amphistomes share definitive hosts, snail hosts, or transmission habitats [51,52]. Co-endemic zones also warrant molecular attention because hybrid, introgressed, or intermediate *Fasciola* forms have been reported where *F. hepatica* and *F. gigantica* overlap [51]. Such overlap can complicate species identification and source attribution; however, direct evidence that climate-driven redistribution is generating new hybrids or altering their pathogenicity remains insufficient.

### 4.3. Taeniids and Environmentally Persistent Helminth Eggs

Taeniid cestodes have received limited attention in climate change parasitology, despite their environmentally persistent eggs and domestic–wildlife transmission cycles [53]. *Echinococcus granulosus* sensu lato circulates mainly between canids and livestock or wild ungulates, whereas *Echinococcus multilocularis* is maintained primarily by wild canids and small mammals, with humans as accidental intermediate hosts [54,55]. Transmission is shaped by climatic effects on egg survival, host movement, predator–prey interactions and domestic–wildlife contact. However, dog roaming, access to infected offal, inadequate deworming, home slaughter, and unsafe carcass disposal may be more important locally than climate [56]. Taeniid eggs generally persist longer under cool, moist and protected conditions, whereas desiccation and solar, including ultraviolet, exposure can reduce viability. Consequently, warming may shorten environmental persistence in dry exposed habitats, while humid or shaded microhabitats may buffer this effect [53,54,55].

In cystic echinococcosis, pastoral mobility, slaughter practices, dog access to offal, wildlife reservoirs, and socioeconomic conditions often dominate local transmission, whereas temperature and moisture modify environmental egg survival [53,54,56]. Therefore, climate should be treated as one component of a wider One Health system rather than a stand-alone driver.

Evidence for the climate-driven redistribution of taeniid cestodes remains limited compared to that of pasture nematodes and snail-borne trematodes. Therefore, climate effects should be interpreted within strongly management-dependent transmission cycles and supported by long-term, integrated surveillance of dogs, livestock, wildlife, environmental egg contamination, and human cases [53,54,56].

### 4.4. Environmentally Transmitted Protozoa

Environmentally transmitted protozoa are climate-sensitive because resistant oocysts and cysts can persist in the soil, feed, and water. Their survival and transport depend on interactions among temperature, moisture, ultraviolet exposure, rainfall, runoff, flooding, and sanitation rather than on temperature alone [24,57,58]. Heavy rainfall can mobilize infective stages from feces and soil into surface waters, whereas dry conditions may concentrate hosts and fecal contamination around scarce water sources [59,60].

For *Toxoplasma gondii*, felids are the definitive hosts, and hydrological transport can connect terrestrial oocyst contamination with livestock, wildlife, and marine ecosystems [24,61]. Precipitation, runoff, erosion, and land-use change may increase oocyst transport from land to water, although these effects should be interpreted alongside felid abundance, farm biosecurity, and watershed connectivity [24,62]. Seropositivity indicates previous exposure but cannot determine when, where or through which route the infection occurred [63].

*Neospora caninum* presents a distinct attribution challenge because endogenous transplacental transmission can maintain infection across generations of cattle independently of short-term environmental variation, whereas dogs and wild canids contribute to horizontal transmission through environmental oocyst contamination [64,65,66]. Climate may modify environmental suitability and canid–livestock interactions; however, its contribution cannot be distinguished without considering reproductive history, maternal infection, herd structure, dog access to feed and placental tissues, and definitive host abundance. Therefore, climate-related associations should be interpreted cautiously and within the broader herd transmission system.

*Cryptosporidium* spp. and *Giardia duodenalis* are important waterborne parasites. Rainfall and runoff can transport oocysts and cysts from livestock, wildlife, and manure-amended land into surface and drinking water sources, whereas droughts may concentrate hosts and contamination around limited water supplies [60,67]. Molecular typing can help identify likely host sources and zoonotic genotypes, but DNA detection alone does not confirm viability or infectivity [58,68,69].

In intensive poultry systems, *Eimeria* transmission is largely governed by the housing microclimate. Litter moisture, ventilation, temperature, and stocking density influence oocyst sporulation and recycling, whereas heat stress can modify host responses and parasite development [70,71,72]. Therefore, adaptation should prioritize housing design, ventilation, litter management, stocking density, and routine environmental monitoring, with geographic risk mapping serving as a complementary tool. External heatwaves can also alter the internal housing microclimate when ventilation or cooling capacity is exceeded, increasing heat stress and intestinal-barrier dysfunction and potentially modifying the severity of *Eimeri* infection rather than simply increasing environmental transmission [72].

Surveillance should combine animal infection data with environmental sampling, molecular source attribution, hydrology, and viability assays, where feasible. This helps distinguish exposure and contamination from active transmission and prevents improved detection from being mistaken for ecological emergence [24,33,69].

### 4.5. Vector-Borne Parasites

Vector-borne parasite transmission is climate-sensitive because temperature and moisture affect the interacting vector–parasite–host systems [20,21]. Temperature influences vector development, survival, activity, and parasite development, whereas humidity and habitat conditions affect vector persistence [19,73]. These responses are nonlinear; for example, moderate warming may extend seasonal activity and promote expansion to higher latitudes or elevations, whereas extreme heat and desiccation may reduce vector survival and habitat suitability [21,73].

For *Babesia* and *Theileria*, climate mainly affects tick distribution and seasonal activity, whereas livestock movement, wildlife–livestock contact, host susceptibility, and tick-control practices influence whether transmission is established [73,74]. Apparent prevalence also varies with sampling effort and diagnostic method because microscopy, serology, and molecular assays measure different epidemiological endpoints [75]. Therefore, a newly detected focus should be considered evidence of possible emergence but not proof of climate-driven expansion. Key vector examples include *Ixodes ricinus* for *Babesia divergens* in Europe, *Rhipicephalus microplus* for *B. bovis* and *B. bigemina* in tropical and subtropical cattle systems, *Hyalomma* spp. for *Theileria annulata*, and *Rhipicephalus appendiculatus* for *T. parva*. In endemic bovine babesiosis systems, a marked reduction in infected-tick challenge can also disrupt enzootic stability and increase clinical risk in susceptible older cattle, although this outcome depends on host immunity, breed, vaccination, and management [73,75].

African animal trypanosomiasis illustrates climate-driven redistribution, rather than uniform amplification. Warming may reduce tsetse abundance in low-elevation areas that exceed their thermal limits, while increasing their abundance at higher elevations or in previously cooler regions [76,77]. Drought and land-use change may further contract or fragment tsetse habitats and concentrate host–vector contact within the remaining suitable areas, including riparian and water-associated vegetation [78]. These patterns represent shifts in vector habitats and transmission interfaces, rather than waterborne transmission. Moreover, mechanical transmission by biting flies and livestock movement can maintain *Trypanosoma vivax* beyond the tsetse fly belt [79]. This focal concentration may be particularly relevant for riverine palpalis-group tsetse, including *Glossina palpalis* and *G. tachinoides*, which depend strongly on riparian habitats; however, drought responses are not uniform across *Glossina* ecological groups [78].

Canine leishmaniasis and dirofilariosis are examples of climate-sensitive transmission in companion animals. Warming may extend sand fly and mosquito activity and increase their suitability in previously marginal areas [80,81,82]. However, dog movement, reservoir availability, local vector habitats, preventive treatment, and vector control coverage remain critical determinants of transmission and geographic establishment [83]. Within biologically suitable thermal ranges, warmer conditions can accelerate *Leishmania* development in phlebotomine sand flies and the development of *Dirofilaria immitis* from microfilariae to infective L3 in mosquitoes, shortening the extrinsic development period before vectors become infectious. This temperature effect interacts with vector survival, humidity, and host availability and is therefore not sufficient on its own to establish transmission [80,81,82,83].

**Figure 2:**
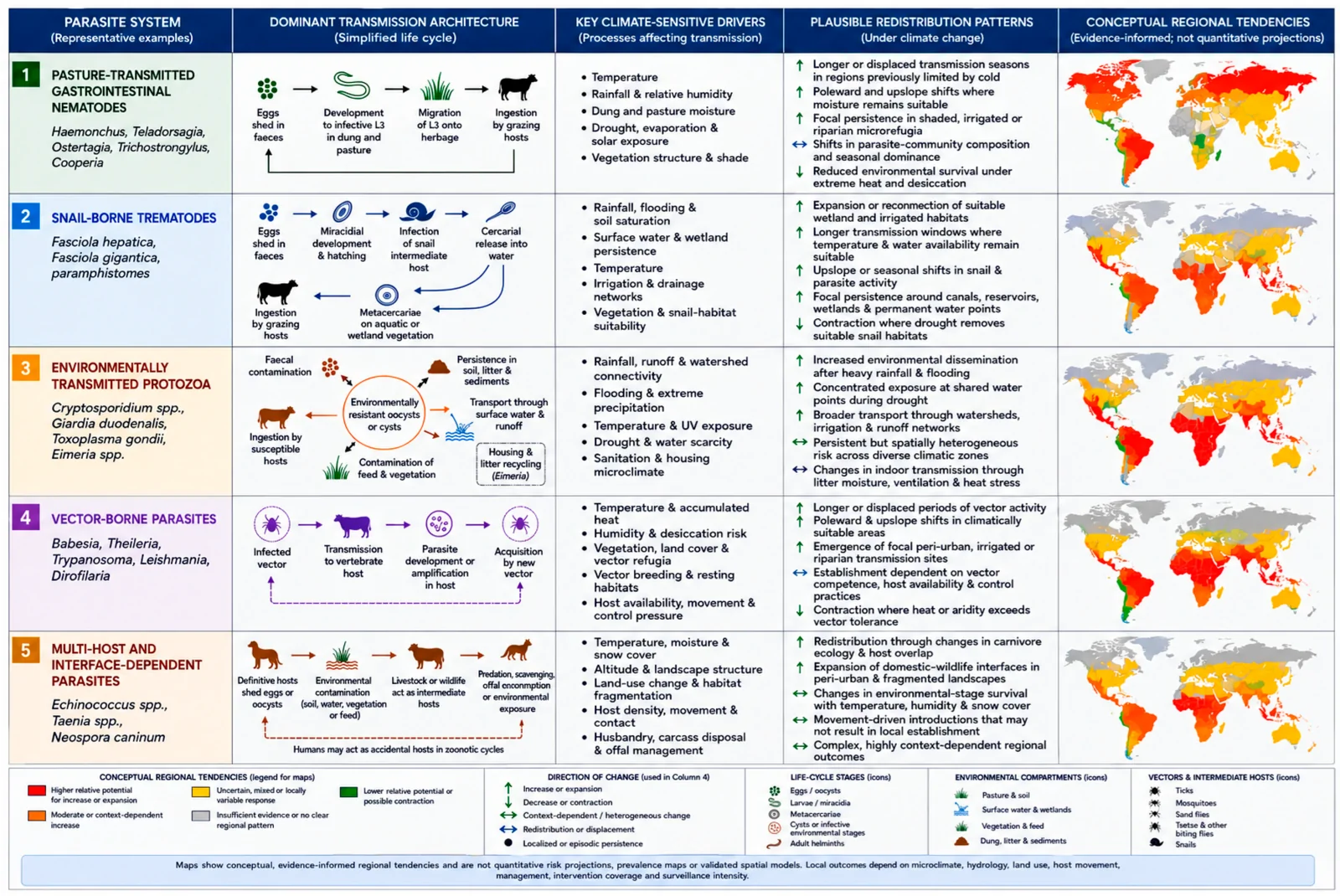
Transmission architectures and conceptual climate-sensitive redistribution patterns of major veterinary parasite systems. The figure compares dominant life cycles, principal climate-sensitive drivers, and plausible changes in geographic range, seasonality, intensity, and host interfaces. World maps summarize evidence-informed conceptual tendencies and should not be interpreted as quantitative predictions or prevalence maps. Local outcomes depend on ecological, management, socioeconomic, and surveillance conditions. In the direction-of-change legend, green upward and downward arrows indicate increase/expansion and decrease/contraction, respectively; green double arrows indicate context-dependent or heterogeneous change, and blue double arrows indicate redistribution, displacement, or other conditional shifts.

In vector-borne systems, the presence of a vector alone does not demonstrate transmission or establishment. Surveillance should integrate vector abundance and infection, vertebrate host infection, animal movement, habitat, microclimate, and intervention coverage [20,73]. Models based solely on macroclimate may misclassify local risk by overlooking host availability, control practices, and microclimatic refugia. Table 2 compares the surveillance requirements of major parasite systems, and Figure 2 illustrates their principal transmission architectures.

## 5. Wildlife–Livestock–Companion Animal Interfaces

Wildlife–livestock interfaces are dynamic transmission landscapes, rather than simple sites of direct contact. Shared pastures, water sources, wetlands, carcasses, vectors, intermediate hosts, and peri-domestic habitats can connect wildlife, livestock, companion animals, and humans through direct or indirect exposure, even in the absence of simultaneous host presence [11,84]. Climate extremes and land-use changes may reorganize these interfaces by altering species distributions, water availability, habitat use, and host movement [31,85].

Wild species may function as reservoir or bridge hosts, spillover recipients, or epidemiological sentinels; however, infection alone does not establish these roles. Reservoir status requires evidence that an epidemiologically connected host population or community can maintain the parasite and transmit it to a defined target population. In contrast, sentinels primarily indicate exposure or local circulation without necessarily contributing substantially to the persistence of parasites [86,87,88]. Similarly, bridge-host status requires evidence that a population links a maintenance community to a target host through ecologically plausible transmission pathways [8]. Robust inference of spillover or spillback therefore requires spatially and temporally coordinated sampling across host populations, supported, where possible, by molecular epidemiology, genomic relatedness, and ecological contact data [89,90]. A practical veterinary example is distinguishing shared exposure from true cross-host transmission of *Haemonchus* spp. and other gastrointestinal nematodes between wild cervids or caprids and domestic ruminants using the same rangelands [18].

Drought can concentrate wildlife, livestock, fecal contamination, and parasite exposure around limited water sources [60]. Conversely, flooding and irrigation may expand or redistribute suitable habitats for snails and mosquitoes and facilitate the transport of environmentally resistant protozoan stages [20,24,26]. Because shared environmental resources may mediate transmission without direct host contact, surveillance should include water, soil, vegetation, feces, vectors, and intermediate hosts, rather than sampling vertebrate hosts alone [18,24].

Free-ranging dogs and cats can connect domestic, wildlife, and human transmission interfaces, although their epidemiological roles vary according to the type of parasite. Dogs are definitive hosts for *Echinococcus* spp., *Neospora caninum*, and *Dirofilaria immitis*, and are the principal domestic reservoir of *Leishmania infantum*, whereas domestic and wild felids are definitive hosts of *Toxoplasma gondii* [53,64,91,92,93]. Their movement among farms, settlements, protected areas, and urban fringes may connect otherwise separate transmission systems. Therefore, interface surveillance should include movement history, preventive treatment, fecal and offal management, and contact with wildlife and vectors [94]. Free-ranging dogs can therefore act as spatial bridges by repeatedly moving across settlement–pasture–wildlife ecotones, carrying ectoparasites and shedding environmentally persistent parasite stages across otherwise separated habitats [53,94].

Zoos, sanctuaries, and rehabilitation centers are managed interfaces where captive wildlife, staff, synanthropic animals, vectors, water, and feed interact. Their veterinary infrastructure and repeated sampling make them useful sentinels for pathogen circulation. However, findings from captive populations should not be extrapolated directly to free-ranging wildlife because husbandry, movement, diet, treatment, and environmental exposure differ substantially [87,95,96].

Operational One Health surveillance should target transmission sites and pathways, rather than host groups alone. Coordinated sampling of livestock, wildlife, companion animals, vectors or intermediate hosts, and environmental matrices, integrated with movement and habitat data, is better suited to identify shared exposures and potential transmission routes than separate sector-specific surveys [97,98]. Co-detection across hosts should be reported strictly as evidence of shared exposure or localized parasite circulation, and should not be interpreted as definitive cross-species transmission unless supported by spatially and temporally linked sampling and molecular or genomic data [88,90].

## 6. Diagnostics, Modeling and Climate-Adaptive Surveillance

### 6.1. Diagnostic Heterogeneity and Surveillance Bias

Apparent parasite redistribution depends not only on parasite ecology but also on surveillance location, host selection, sampling intensity, and the diagnostic endpoint used [11,99]. Improved assays and shifts from conventional to species-resolved molecular methods may reveal previously undetected infections, whereas uneven sampling, increased awareness, and changes in reporting can create the appearance of ecological emergence [99,100]. Therefore, surveillance and diagnostic design should be considered part of the attribution problem rather than secondary methodological details [33].

Diagnostic methods measure distinct biological endpoints—including previous exposure, active infection, parasite shedding, burden, environmental contamination, and molecular detection—and should not be interpreted interchangeably [58,68,101]. Serological responses may persist after infection, whereas coprological methods may miss prepatent, intermittent, or low-intensity infections [101]. Similarly, PCR can detect parasite DNA without confirming its viability or infectivity [69]. Epidemiological models should therefore use diagnostic endpoints that match the transmission question and explicitly report assay sensitivity, specificity, target stage, and interpretation.

Longitudinal and standardized surveillance is needed to distinguish persistent parasite redistribution from seasonal variations and changes in management, host movement, or diagnostic efforts. Wildlife data are especially prone to accessibility and selection biases because they often rely on opportunistic samples such as hunted animals, roadkill, carcasses, rehabilitation cases, or feces [11,18,99].

### 6.2. Molecular and Environmental Surveillance

PCR, qPCR, digital PCR, metabarcoding, and next-generation sequencing can improve parasite detection, quantification, species resolution, mixed infection characterization, and resistance surveillance [100,102,103]. However, molecular results should address a predefined attribution question and be interpreted alongside epidemiological and ecological evidence because nucleic acid detection alone does not demonstrate parasite viability, source, or active transmission.

However, molecular findings require careful interpretation. Parasite DNA detection alone does not establish viability, infectivity, reservoir status, or clinical relevance [69,88]. Results may also be influenced by incomplete reference databases, primer bias, assay sensitivity, and limited methodological standardization [104,105]. Molecular evidence should therefore be interpreted alongside epidemiological, ecological, and, where relevant, viability or infectivity data.

Environmental DNA is promising for detecting aquatic parasites, snail intermediate hosts, vector larvae and contaminated water. However, its detection and spatial interpretation vary with temperature, ultraviolet exposure, water chemistry, microbial degradation, sediment dynamics, hydrology, and sampling design [105,106,107,108]. Field assays should therefore be locally validated against direct sampling of parasites and relevant hosts and interpreted alongside evidence of viability and active transmission, particularly in warm and hydrologically dynamic tropical systems [105].

To support global applicability, we propose a tiered, resource-sensitive surveillance framework. Tier 1 uses affordable field methods to detect credible geographic, seasonal, intensity, or interface signals; Tier 2 confirms these signals and characterizes likely sources at regional laboratories; and Tier 3 applies advanced molecular and genomic tools in reference laboratories to resolve cryptic species, environmental pathways, and resistance mechanisms [97,100,109]. The proposed escalation triggers are precautionary and evidence-informed rather than prospectively validated thresholds. They are based on the unusualness, persistence, discordance, and potential animal-health, public-health, or conservation consequences of a signal, together with the need for confirmatory resolution [97,100,109,110]. Escalation to a higher analytical tier increases diagnostic resolution but does not, by itself, increase confidence in climate attribution; attribution still requires convergence across longitudinal, ecological, epidemiological, and diagnostic evidence. Table 3 summarizes the proposed tools, outputs, escalation criteria, and safeguards for affordability, accessibility, quality assurance, specimen referral, and data sharing.

### 6.3. Spatial, Mechanistic and Machine-Learning Models

GIS and remote sensing can integrate parasite occurrence records with climate, vegetation, elevation, land cover, hydrology, soil, host distribution, and management, enabling repeated environmental assessments across large areas [110,111,112]. Their value depends on matching the spatial and temporal resolution of environmental data with parasite biology and transmission pathways.

Risk maps may reflect sampling effort rather than ecological suitability when occurrence records are spatially clustered or biased toward accessible areas. Presence-only data, coarse environmental layers, positional uncertainty, spatial autocorrelation, and projection into non-analog climates may reduce model transferability and create false precision [113,114,115]. Spatially and temporally independent validation and explicit uncertainty estimates are therefore essential [116].

Correlative models are useful for identifying potential risk areas, whereas mechanistic models represent processes such as parasite development, mortality, vector activity, hydrology, and snail habitat. Mechanistic models can strengthen biological interpretation, but require detailed parameters that remain unavailable for many parasites and regions [45]. Hybrid approaches should therefore be evaluated according to predictive performance, biological plausibility, transferability, uncertainty, and usefulness for decision-making [110,116].

### 6.4. Early Warning and One Health Data Integration

Early warning systems should integrate standardized parasitological data with microclimate, hydrology, land use, host movement, and management data to identify operational thresholds and emerging transmission signals. The proposed tiered framework in Table 3 provides a structured basis for escalation from affordable field observations to regional confirmation or reference-level analysis according to the strength and potential consequences of a signal [97,112]. These escalation rules remain provisional and require prospective validation. Models should be externally validated across regions and diagnostic settings, with prediction uncertainty and data limitations explicitly reported [113,115].

**Figure 3:**
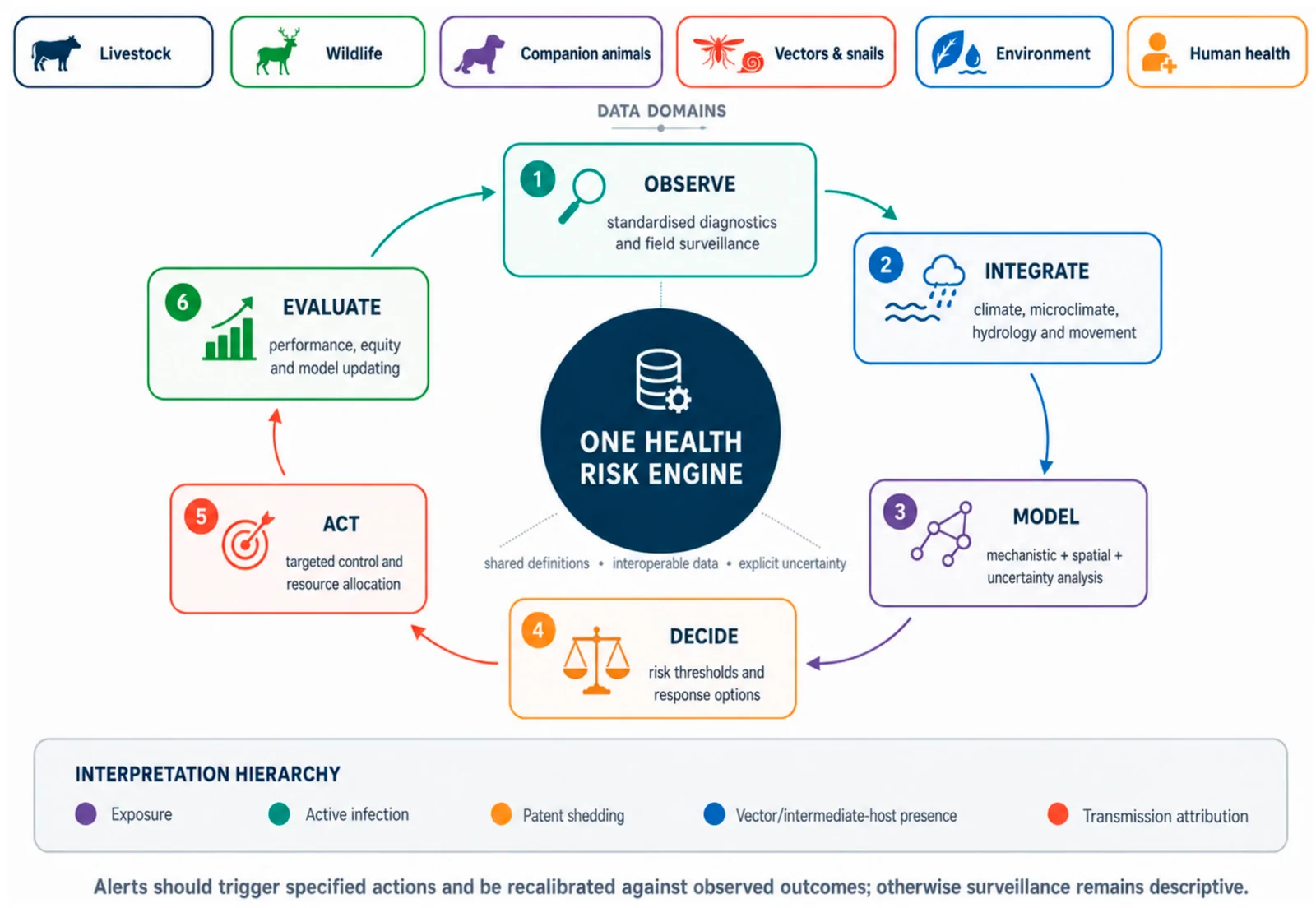
Proposed climate-adaptive One Health surveillance cycle. Standardized detection is integrated with climate, microclimate, hydrology, movement and management data. The proposed tiered diagnostic structure in Table 3 suggests when evidence may progress from field screening to regional confirmation or reference-level analysis. Forecasts are linked to predefined actions and recalibrated based on observed outcomes. The cycle is conceptual and requires prospective validation.

Alerts should be linked to predefined actions, responsible institutions, evidence thresholds, diagnostic tiers and evaluation criteria. The proposed observe–integrate–model–decide–act–evaluate cycle shown in Figure 3 also requires interoperable data, cross-sectoral information-sharing agreements, and equitable access to laboratory and analytical capacity [97,109]. Advanced tools should complement, rather than replace, field epidemiology, microscopy, or local ecological knowledge. Table 4 summarizes the main evidence gaps and proposed minimum priorities for improvement in the field.

## 7. Climate-Adaptive Control and One Health Implementation

### 7.1. From Fixed Calendars to Risk-Based Control

Fixed treatment calendars assume predictable transmission; however, warming, irregular rainfall, drought, flooding, and other extreme events can shift larval availability, snail activity, vector abundance, and host exposure. Historical schedules may consequently promote treatment during low-risk periods or fail to cover displaced transmission peaks [17,18,21,25]. Risk-based control combines current infection indicators with environmental and management information to guide the timing and intensity of interventions.

Targeted selective treatment uses parasitological, clinical, or production indicators, including fecal egg counts, FAMACHA scores, body condition, weight gain, milk production, and clinical signs to identify animals that are most likely to benefit from treatment [117,118]. By leaving part of the parasite population unexposed to treatment, these approaches maintain refugia that may dilute resistant genotypes, although suitable indicators and the required proportion of untreated parasites vary among host–parasite systems [119,120]. We propose a climate–refugia paradox: severe drought or heat may reduce free-living parasite stages and environmental refugia; therefore, whole-group treatment during these periods could expose a large proportion of the remaining parasite population to drug selection [119,121]. However, maintaining refugia should not compromise animal welfare. Treatment decisions should therefore balance individual clinical need with recent rainfall, pasture and fecal moisture, larval availability, the proportion of untreated hosts, and post-treatment efficacy testing [119,120].

### 7.2. Resistance-Aware Helminth Management

Climate may indirectly influence anthelmintic resistance by altering the development, transmission duration, treatment frequency, and refugia size of parasites. Longer transmission seasons may increase treatment demand, whereas droughts can reduce environmental refugia and intensify selection when treatment is applied. However, direct field evidence linking climate change to resistance evolution remains limited [119,120,122], and the proposed climate–refugia hypothesis requires prospective empirical testing.

Resistance-aware control should confirm the need for treatment, preserve untreated animals when welfare permits, and verify efficacy through fecal egg count reduction testing [119,120,123]. Suspected failure may require species-level or molecular investigation [100], whereas accurate dosing, appropriate drug selection, quarantine, justified combinations, and biosecurity remain essential [122,124].

Grazing management should consider rainfall, larval survival, pasture conditions, irrigation, stocking density and rest periods. Mixed-species or rotational grazing may reduce host-specific parasites, but effectiveness varies among production systems; therefore, local monitoring is preferable to fixed recommendations [22,37,45,125]. Where host specificity is strong, mixed grazing can dilute pasture contamination because larvae ingested by a non-susceptible host fail to establish. However, this biological dilution or “vacuum-cleaner” effect is not universal because some gastrointestinal nematodes can be shared across ruminant hosts; local parasite composition should therefore guide its use [125].

### 7.3. Habitat, Water and Vector Management

Water management is important for controlling trematodes and waterborne protozoa. Drought may concentrate animals at shared water sources, whereas heavy rainfall and flooding can expand snail habitats or transport oocysts and cysts via runoff. These risks support protecting drinking points, reducing fecal contamination, limiting access to high-risk wetlands, and increasing surveillance after extreme rainfall [24,25,26,58]. Where feasible, fencing livestock away from seasonal snail habitats and providing protected off-site water can reduce contact with *Fasciola* transmission microrefugia and contaminated surface water. These measures reduce exposure rather than guarantee interruption of transmission and require protection of troughs from fecal contamination [24,25,26,58].

Climate-informed vector control should follow current vector activity rather than historical endemicity. Priorities include tick and mosquito surveillance, habitat management, sustained heartworm prevention where transmission risk persists, and canine testing and repellents in areas at risk of leishmaniasis [19,80,82,126].

### 7.4. Interface-Sensitive and Companion-Animal Interventions

Interventions at wildlife–livestock interfaces should balance disease prevention with ecological integrity. Measures that restrict host movement or modify access to shared resources should follow surveillance that clarifies whether wildlife maintains transmission, connects host populations, experiences spillover, or acts as a sentinel, because each role requires a different response [11,127].

Dogs and cats may serve as definitive hosts, reservoirs, bridge hosts, sentinels, or sources of environmental contamination for several zoonotic and vector-borne parasites. Control measures should combine diagnosis, preventive treatment, vector protection, feces management, responsible ownership, and context-specific education. Working dogs in pastoral systems and pets in peri-urban areas should therefore be considered distinct epidemiological groups [24,94,128].

### 7.5. Governance, Adoption and Equity

**Figure 4:**
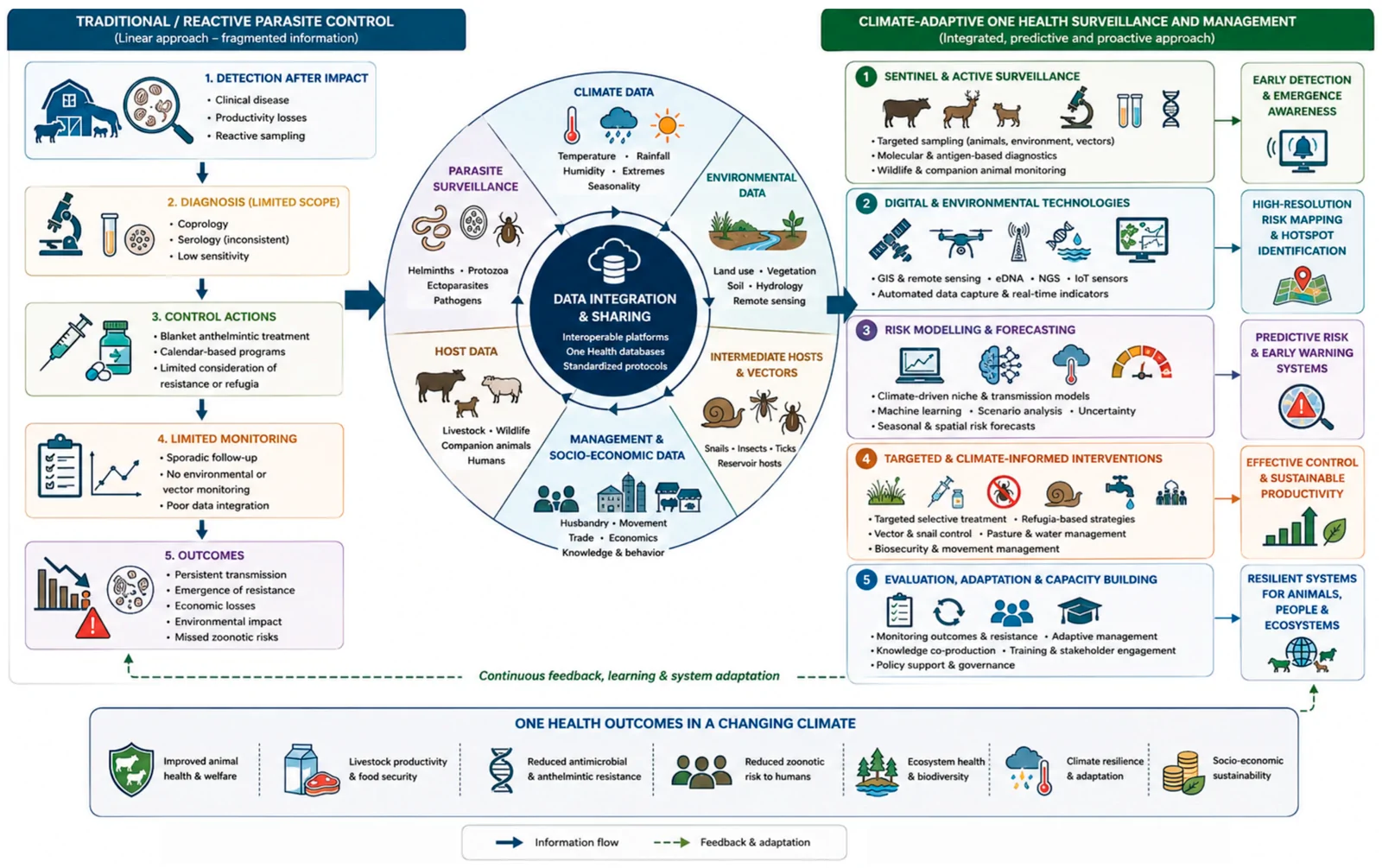
Transition from reactive parasite control to climate-adaptive One Health surveillance and management. Traditional control commonly begins after clinical or production impacts and relies on fragmented diagnostics, calendar-based interventions, and limited follow-up. The proposed climate-adaptive system integrates parasite, host, vector, intermediate-host, environmental, climatic, and socioeconomic data to support active surveillance, risk forecasting, targeted intervention, evaluation, and continuous adaptation. Arrows indicate the principal relationships within the framework: solid blue arrows represent the main information flow and data integration, whereas dashed green arrows indicate continuous feedback, learning, and system adaptation. The short colored arrows connecting the five climate-adaptive domains with their corresponding outcome boxes are visual connectors and do not denote additional process categories.

Climate-adaptive management requires clear and coordinated responsibilities across veterinary services, wildlife agencies, meteorological institutions, water managers, laboratories, and public health systems. Adoption depends on practical thresholds, transparent benefits and costs, co-design with users, and local validation. Equity should be treated as a measure of system performance: the tiered framework in Table 3 links affordable field methods with shared regional and reference capacity, rather than assuming universal access to advanced technology. Figure 4 integrates these principles by contrasting traditional reactive parasite control with a climate-adaptive One Health system based on surveillance, data integration, risk forecasting, targeted interventions, outcome evaluations, and continuous adaptations.

## 8. Limitations of the Evidence Base and Research Priorities

Both the available evidence and this review have important limitations. The literature is geographically and methodologically uneven, with temperate livestock systems overrepresented and tropical, subtropical, wildlife, and extensive pastoral systems remaining under-surveilled. Studies also frequently examine livestock, wildlife, companion animals, vectors, intermediate hosts, and environmental stages separately, limiting the reconstruction of complete transmission systems. Because this is a critical narrative review, the literature selection was not exhaustive, and no formal risk of bias assessment, certainty grading, or quantitative synthesis was undertaken. The proposed attribution framework should therefore be regarded as a conceptual and operational decision aid rather than a validated causal instrument.

Much of the available evidence is cross-sectional and based on annual climatic averages, which cannot reliably distinguish persistent redistribution from seasonal variability, land-use change, animal movement, management, treatment history, or improved detection. Stronger attribution will require standardized longitudinal surveillance, biologically relevant measurements of microclimate and hydrology, explicit counterfactuals and models validated using independent data.

Research priorities include prospectively testing the four redistribution dimensions and five attribution filters, integrating host, vector, intermediate host, and environmental sampling at selected interfaces, validating diagnostic escalation and predictive models against transmission outcomes, and determining whether climate-informed decisions reduce disease, unnecessary treatment, resistance selection, and economic loss. Implementation research must also address affordability, training, interpretability, data governance, benefit-sharing, and sustained financing.

## 9. Conclusions

Climate change does not uniformly increase veterinary parasitism; rather, it redistributes transmission opportunities across geographic areas, seasons, infection intensity, and host–environment interfaces. The proposed framework evaluates these signals through five attribution domains: environmental suitability, parasite life-cycle response, vector or intermediate host response, host-interface change, and surveillance artifacts. This structure helps prevent new detections, altered burdens, or cross-species signals from being prematurely labeled as climate-driven.

Because transmission architectures differ, surveillance and control must be mechanism-specific. Tiered systems should connect affordable field indicators and local environmental observations with regional confirmation and advanced molecular analyses when justified. Risk-based interventions should complement fixed calendars and, where locally validated, may progressively replace rigid schedules while accounting for animal welfare, refugia, water and habitat conditions, vector or snail activity, and wildlife–livestock–companion-animal interfaces. The framework now requires prospective validation across different climates and production systems. Its practical value should ultimately be judged by whether it improves decisions and reduces disease, unnecessary treatment, resistance selection, economic losses, and risks to biodiversity and to public health.

## Figures and Tables

**Table 1:** Operational attribution and diagnostic decision matrix for climate-sensitive redistribution of parasites.

Dimension	Operational Signal	Five-Way Attribution Checks	Minimum Evidence	False Signal Avoided
Geographic	Expansion, contraction, or displacement of the observed range	Local suitability; parasite-stage response; vector/snail response; host movement; stable detection effort	Repeated georeferenced sampling, comparable diagnostics, microhabitat and movement data, local transmission evidence	A new assay, imported infection, or intensified surveillance mistaken for range expansion
Seasonal	Earlier onset, later end, altered peak, or overwintering	Weather–stage coherence; vector/snail phenology; host exposure; stable sampling calendar and assay	Multi-season data with stage-specific endpoints and contemporaneous microclimate or hydrology	Irregular sampling, treatment timing, or a single anomalous season mistaken for persistent change
Intensity	Change in incidence, burden, shedding, vector infection, or impact	Climate dose–response; host structure; treatment and management; assay sensitivity; environmental abundance	Quantitative denominators, burden/incidence, treatment history, and environmental or vector measurements	More sensitive testing, herd composition, or treatment failure mistaken for intensified transmission
Interface	New or intensified circulation among hosts or environments	Shared habitat; host contact/movement; vector or snail bridge; molecular relatedness; balanced multi-host sampling	Concurrent host, vector/environmental, and ecological contact data; molecular typing where feasible	Shared seropositivity or isolated detections mistaken for spillover or reservoir status

**Table 2:** Climate-sensitive transmission architectures, plausible redistribution patterns, and priority surveillance variables across selected representative veterinary parasite systems.

Parasite System	Transmission Pathway	Principal Climate and Contextual Drivers	Plausible Redistribution Pattern	One Health Relevance	Priority Surveillance
Gastrointestinal nematodes: *Haemonchus*, *Teladorsagia*, *Ostertagia*, *Trichostrongylus*, *Cooperia*	Eggs and larvae develop in feces and pasture; infective L3 migrate to herbage	Temperature, rainfall, humidity, pasture moisture, drought, shade	Drought–rainfall pulses and microrefugia in tropical systems; longer seasons in some temperate areas; contraction where heat and desiccation exceed larval tolerance	Livestock productivity, anthelmintic resistance, wildlife–livestock parasite sharing	Fecal egg counts, FAMACHA/body condition, species resolution, pasture/dung moisture, rainfall pulses, larval monitoring, and treatment records
Snail-borne trematodes: *Fasciola hepatica*, *F. gigantica* and paramphistomes	Snail-borne transmission through aquatic/semi-aquatic habitats and metacercariae on vegetation	Rainfall, soil moisture, flooding, irrigation, temperature, water permanence	Expansion or reconnection of wet habitats; persistence in irrigation and water-point refuges; post-flood pulses; altitudinal or seasonal shifts	Livestock losses, zoonotic fasciolosis, wetland-associated transmission	Coprology/coproantigen/serology, snail mapping and infection, water-network and flood history, eDNA where validated, and animal access to water
Lungworms: *Dictyocaulus viviparus*, *Dictyocaulus filaria*, protostrongylids	Larvae develop on pasture; some use gastropod intermediate hosts	Rainfall, humidity, temperature, pasture moisture, grazing season	Altered outbreak timing; increased unpredictability in temperate grazing systems; wildlife–livestock overlap	Respiratory disease, production loss, wildlife health	Larval Baermann, clinical surveillance, pasture infectivity models, vaccination history
Taeniid cestodes: *Echinococcus granulosus sensu lato*	Eggs shed by canids contaminate environment; livestock/wildlife ingest eggs	Temperature, humidity, snow cover, land use, host movement	Changes in egg survival and domestic–wild cycle overlap; expansion through dog/livestock movement	Cystic echinococcosis, food safety, public health	Dog surveillance, abattoir data, livestock cyst inspection, wildlife monitoring, molecular typing
*Toxoplasma gondii*	Felids shed oocysts; oocysts persist in soil, water, feed and food chains	Rainfall, runoff, humidity, temperature, flooding, land–sea flow	Greater waterborne and foodborne dissemination after rainfall/flooding; watershed-mediated spread	Food safety, reproductive losses, wildlife/marine mammal health, human toxoplasmosis	Felid ecology, serology, oocyst detection, water/soil sampling, watershed modeling
Waterborne protozoa: *Cryptosporidium* spp., *Giardia duodenalis*	Oocysts/cysts contaminate water, soil, manure, feed and runoff systems	Rainfall, flooding, drought, runoff, turbidity, water temperature	Increased contamination after heavy rainfall; focal exposure during drought at shared water points	Zoonotic diarrhoeal disease, calf/lamb morbidity, water safety	Water sampling, molecular typing, livestock/wildlife source tracking, hydrological monitoring
Tick-borne protozoa: *Babesia bovis*, *B. bigemina*, *Theileria annulata*, *T. parva*, *T. orientalis*	Transmission through ixodid ticks feeding on domestic and wild hosts	Temperature, humidity, vegetation, tick survival, host availability	Longer or displaced vector seasons; upward/poleward shifts; contraction in overheated areas; focal persistence in riparian, irrigated, or peri-domestic refuges	Livestock mortality, anemia, production losses, wildlife reservoirs	Vector abundance/infection, host movement, riparian and irrigation habitat, livestock and wildlife surveillance, confirmation, and intervention coverage
Tsetse-transmitted trypanosomes: *Trypanosoma congolense*, *T. vivax*, *T. brucei*	Tsetse flies transmit parasites between livestock and wildlife reservoirs	Temperature, vegetation, humidity, land cover, host movement	Decline in overheated lowlands; increased suitability in cooler/highland areas; shifting risk corridors	African animal trypanosomiasis, rural livelihoods, wildlife reservoirs	Tsetse trapping, livestock parasitemia, molecular diagnosis, land-cover and climate modeling
Sand fly-borne *Leishmania infantum*	Sand flies transmit parasite among dogs, wildlife reservoirs and humans	Temperature, humidity, land cover, peri-domestic microhabitats	Northward/altitudinal expansion in Europe and other suitable regions; longer vector seasons	Canine leishmaniosis, human visceral leishmaniasis, companion-animal sentinel role	Dog serology/PCR, sand fly surveillance, reservoir studies, climate suitability mapping
Mosquito-borne filariae: *Dirofilaria immitis*, *D. repens*	Mosquitoes transmit larvae; development depends on accumulated heat	Temperature, mosquito season length, rainfall, urban water, dog movement	Longer transmission windows; emergence in previously marginal areas; companion-animal travel effect	Canine heartworm, zoonotic subcutaneous dirofilariosis	Dog testing, mosquito monitoring, degree-day models, preventive treatment records
Multi-host parasites at wildlife–livestock interfaces	Shared water, pasture, vectors, intermediate hosts, carcasses, soil and peri-domestic environments	Drought, flooding, heatwaves, habitat fragmentation, resource sharing	Increased spillover/spillback where climate stress increases contact; changing bridge-host roles	One Health risk, conservation, livestock productivity, zoonotic exposure	Interface-based sampling, GPS/camera traps, molecular typing, shared environmental surveillance

**Table 3:** Proposed tiered diagnostic surveillance framework for climate-sensitive veterinary parasitology.

Tier	Core Tools and Observations	Information Produced	Proposed Escalation Trigger	Safeguards
Tier 1: field/low cost	McMaster or other standardized fecal egg counts; flotation, sedimentation, or Baermann; FAMACHA, body condition and production; basic vector/snail inspection; local rainfall, temperature, pasture/dung moisture and water-point records	Patent shedding, syndromic burden, treatment need, and local environmental context	Unexpected spatial/seasonal signal; high morbidity; treatment failure; new interface or vector/snail observation	Simple SOPs, training, QA samples, denominators, harmonized metadata; a negative low-sensitivity test is not absence
Tier 2: regional/confirmatory	Serology, coproantigen, PCR/qPCR, fecal egg-count reduction testing, larval culture, vector/snail xenomonitoring, targeted water/habitat sampling, cluster analysis	Exposure or active infection, species/group resolution, efficacy, infected vectors/snails, and regional hotspots	Discordant field/lab results; suspected expansion; persistent transmission; zoonotic or conservation relevance	Match assay to biological question; report endpoint and performance; use shared regional infrastructure
Tier 3: reference/research	Digital PCR, metabarcoding or nemabiome sequencing, validated eDNA, targeted or whole-genome sequencing, resistance alleles, genomic epidemiology, mechanistic attribution models	Cryptic diversity, mixed infection, environmental pathways, connectivity, resistance mechanisms, and higher-resolution evidence relevant to attribution	Novel or complex event; cross-border spread; failed control; unexplained emergence; diagnostic/model validation need	Contamination controls, curated databases, uncertainty, external QA, and transparent data governance
Cross-tier integration	Archived samples; interoperable identifiers; common climate, microclimate, host, movement, treatment and land-use metadata; predefined alerts and field feedback	Comparable longitudinal evidence and an auditable chain from detection to action	Any unresolved alert or response with major animal, public-health, or ecological consequences	Advanced tools complement rather than displace field capacity; escalation must be timely, affordable, and actionable

**Table 4:** Principal evidence limitations and minimum priorities for climate-sensitive veterinary parasitology.

Domain	Current Limitation and Attribution Risk	Proposed Minimum Priority	Most Relevant Systems
Causal attribution and longitudinal evidence	Cross-sectional climate associations may reflect seasonality, land use, animal movement, treatment, detection effort, or reporting, rather than persistent redistribution.	Establish repeated standardized surveillance across climate gradients, integrate competing drivers, and test explicit counterfactuals.	All groups
Microclimate, hydrology, vectors and intermediate hosts	Broad climate layers omit dung humidity, shade, water permanence, snail habitats, vector resting sites, and other conditions that regulate transmission.	Combining ground measurements, remote sensing, habitat mapping, and vector/snail abundance and infection.	Free-living helminths, trematodes, vector-borne parasites, environmental protozoa
Diagnostic comparability and environmental molecular tools	Different assays measure exposure, active infection, shedding, DNA, or contamination; however, eDNA or PCR positivity may not establish viability or transmission.	Standardize reporting, validate assay performance and eDNA against biological endpoints, and apply a tiered diagnostic framework.	All groups, especially cryptic and environmental infections
Multi-host and One Health integration	Livestock, wildlife, companion animals, vectors, intermediate hosts, and environments are usually sampled separately, obscuring the roles of reservoirs, bridge hosts, and spillovers.	Concurrent interface-based sampling, movement/contact data, and molecular typing were used where informative.	Zoonotic and multi-host parasites
Predictive modeling and early warning	Correlative models can amplify sampling bias, diagnostic errors, scale mismatches, and non-analog climate projections.	Combining mechanistic and statistical models, external validation, uncertainty mapping, and predefined action thresholds.	All climate-sensitive systems
Equity, implementation and capacity	Tropical and subtropical regions are under-surveilled, and technology-intensive proposals may be unaffordable or impractical.	Strengthening field diagnostics, regional laboratory networks, training, participatory surveillance, interoperable data, and sustained financing.	All groups

## Data Availability

No new data were created or analyzed in this study. Data sharing is not applicable to this article.
